# Multiparametric MRI may Help to Identify Patients With Prostate Cancer in a Contemporary Cohort of Patients With Clinical Bladder Outlet Obstruction Scheduled for Holmium Laser Enucleation of the Prostate (HoLEP)

**DOI:** 10.3389/fsurg.2021.633196

**Published:** 2021-02-25

**Authors:** Mike Wenzel, Maria N. Welte, Lina Grossmann, Felix Preisser, Lena H. Theissen, Clara Humke, Marina Deuker, Simon Bernatz, Philipp Gild, Sascha Ahyai, Pierre I. Karakiewicz, Boris Bodelle, Luis A. Kluth, Felix K. H. Chun, Philipp Mandel, Andreas Becker

**Affiliations:** ^1^Department of Urology, University Hospital Frankfurt, Goethe University Frankfurt am Main, Frankfurt, Germany; ^2^Cancer Prognostics and Health Outcomes Unit, Division of Urology, University of Montréal Health Center, Montréal, QC, Canada; ^3^Department of Diagnostic and Interventional Radiology, University Hospital Frankfurt, Frankfurt am Main, Germany; ^4^Department of Urology, University Hospital Hamburg-Eppendorf, Hamburg, Germany; ^5^Department of Urology, University Hospital Goettingen, Goettingen, Germany

**Keywords:** HOLEP, fusion biopsy, systematic biopsy, PSA, IPSS, BPH, BPO

## Abstract

**Objective:** To investigate the value of standard [digital rectal examination (DRE), PSA] and advanced (mpMRI, prostate biopsy) clinical evaluation for prostate cancer (PCa) detection in contemporary patients with clinical bladder outlet obstruction (BOO) scheduled for Holmium laser enucleation of the prostate (HoLEP).

**Material and Methods:** We retrospectively analyzed 397 patients, who were referred to our tertiary care laser center for HoLEP due to BOO between 11/2017 and 07/2020. Of those, 83 (20.7%) underwent further advanced clinical PCa evaluation with mpMRI and/or prostate biopsy due to elevated PSA and/or lowered PSA ratio and/or suspicious DRE. Logistic regression and binary regression tree models were applied to identify PCa in BOO patients.

**Results:** An mpMRI was conducted in 56 (66%) of 83 patients and revealed PIRADS 4/5 lesions in 14 (25%) patients. Subsequently, a combined systematic randomized and MRI-fusion biopsy was performed in 19 (23%) patients and revealed in PCa detection in four patients (5%). A randomized prostate biopsy was performed in 31 (37%) patients and revealed in PCa detection in three patients (4%). All seven patients (9%) with PCa detection underwent radical prostatectomy with 29% exhibiting non-organ confined disease. Incidental PCa after HoLEP (*n* = 76) was found in nine patients (12%) with advanced clinical PCa evaluation preoperatively. In univariable logistic regression analyses, PSA, fPSA ratio, and PSA density failed to identify patients with PCa detection. Conversely, patients with a lower International Prostate Symptom Score (IPSS) and PIRADs 4/5 lesion in mpMRI were at higher risk for PCa detection. In multivariable adjusted analyses, PIRADS 4/5 lesions were confirmed as an independent risk factor (OR 9.91, *p* = 0.04), while IPSS did not reach significance (*p* = 0.052).

**Conclusion:** In advanced clinical PCa evaluation mpMRI should be considered in patients with elevated total PSA or low fPSA ratio scheduled for BOO treatment with HoLEP. Patients with low IPSS or PIRADS 4/5 lesions in mpMRI are at highest risk for PCa detection. In patients with a history of two or more sets of negative prostate biopsies, advanced clinical PCa evaluation might be omitted.

## Introduction

Holmium laser enucleation of the prostate (HoLEP) remains the current standard of care in the treatment of bladder outlet obstruction (BOO) due to benign prostate hyperplasia (BPH) patients with severe lower urinary tract symptoms (LUTS) after failure of pharmacological treatment (1). Since for both BPH and prostate cancer (PCa) elderly men account for the majority of patients and age represents a major risk factor, cancerous lesions may be present in BPH patients (2–5). Nonetheless, if PCa diagnoses would change the management of BPH, EAU guidelines recommend prostate-specific antigen (PSA) testing prior to BPH treatment (1). Moreover, prostate biopsy before BPH treatment is related to lower risks of incidental PCa (6). However, standard clinical PCa evaluation using digital rectal examination (DRE) and PSA testing may be of limited value due to confounding factors often observed in patients with clinical BOO, such as prostate enlargement, urinary retention, or presence of an indwelling transurethral catheter. Interestingly, in the contemporary literature, little is known about PCa detection rates of advanced clinical PCa evaluation using multiparametric MRI (mpMRI) or prostate biopsies in patients with clinical BOO.

To address this void, we relied on our institutional database. We aimed to investigate the efficacy of standard (DRE, PSA) and advanced (mpMRI, prostate biopsy) clinical evaluation for PCa detection in patients with clinical BOO scheduled for HoLEP.

## Materials and Methods

### Study Population

**Figure 1:**
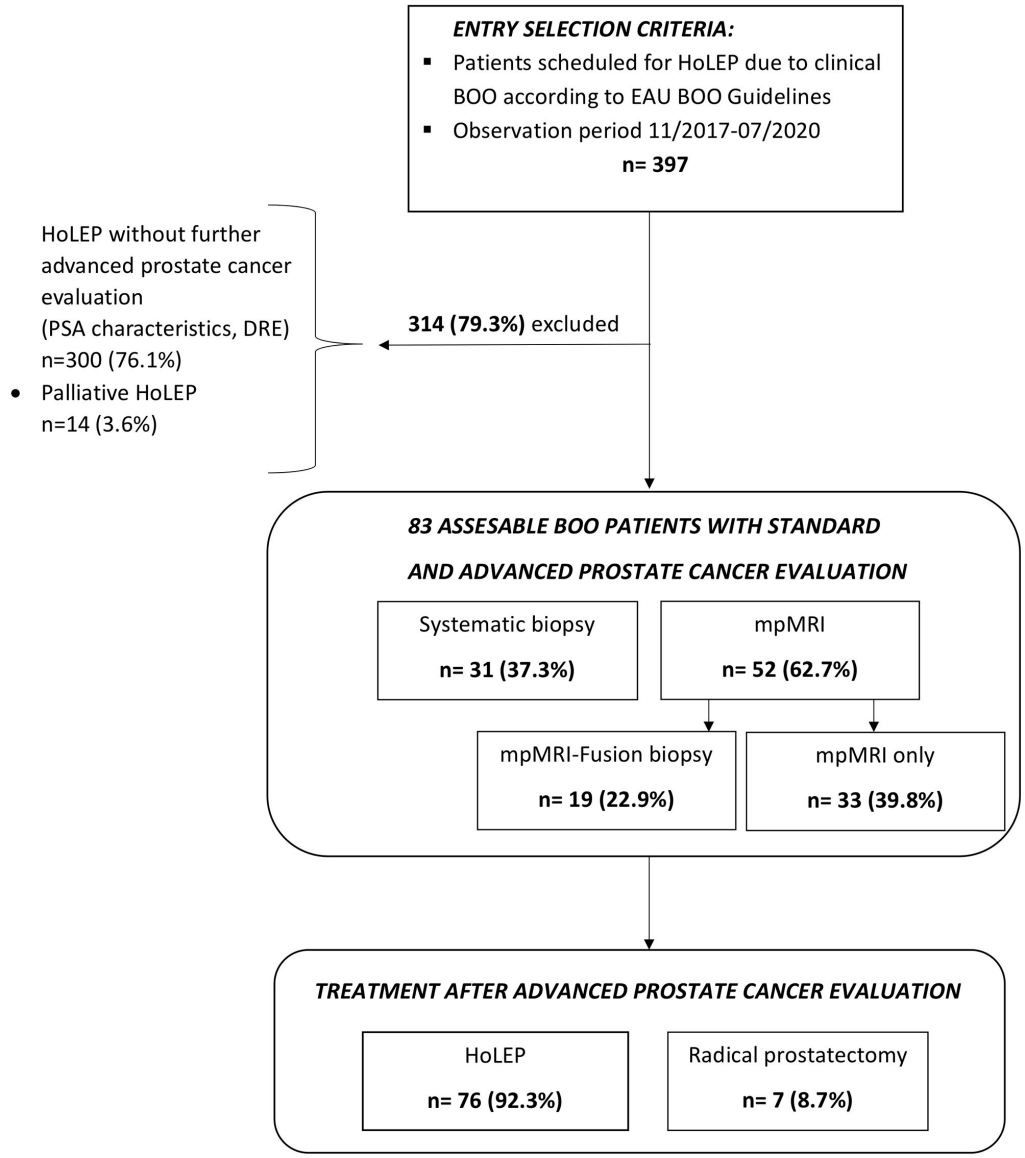
CONSORT (Consolidated Standards of Reporting Trials) diagram of 397 patients with clinical Bladder Outlet Obstructive (BOO) symptoms and additional standard and advanced prostate cancer evaluation prior to scheduling for Holmium Laser Enucleation of the Prostate (HoLEP) between 11/2017 and 07/2020.

After approval of the ethics committee, all patients who were referred for HoLEP due to clinical BOO to our tertiary care laser center at Department of Urology, Frankfurt University Hospital, between 11/2017 and 07/2020 were consecutively identified in the institutional database and evaluated retrospectively (*n* = 397). All patients underwent standard clinical PCa evaluation using DRE and PSA testing [total PSA and free PSA (fPSA)/total PSA ratio (fPSA ratio)]. After exclusion of patients not undergoing further advanced clinical PCa evaluation (*n* = 300) and of patients undergoing palliative HoLEP (*n* = 14), our final study cohort consisted of 83 patients (Figure 1). These 83 (20.7%) patients underwent advanced clinical PCa evaluation based on suspicious prostate characteristics in previous examinations, such as elevated total PSA level and/or lowered fPSA ratio and/or suspicious digital rectal examination (DRE). Clinical advanced PCa evaluation was defined as either multiparametric magnetic resonance imaging (mpMRI) or systematic biopsy or MRI-fusion biopsy of the prostate within 1 year prior to HoLEP. All mpMRI examinations were primarily performed and read by an experienced radiologist and confirmed by a board-certified radiologist.

### Statistical Analysis

Descriptive statistics included frequencies and proportions for categorical variables. Means, medians, and interquartile ranges (IQR) were reported for continuously coded variables. The Chi-square test was used for statistical significance in proportions' differences. The *t*-test and Kruskal–Wallis test examined the statistical significance of means' and distributions' differences.

First, to investigate the characteristics of patients with PCa detection, the cohort was stratified by prostate biopsy status into patients with positive prostate biopsy (“PCa detection”), and patients with negative biopsy status or with unsuspicious mpMRI (“Negative PCa detection”). The strata were compared accordingly.

Second, univariable and multivariable logistic regression models were fitted to predict positive prostate biopsy. Moreover, to better display the relation between prostate cancer detection in BPH patients with clinical BOO, a binary regression tree was fitted, as previously described, using PIRADS score, fPSA ratio, age, and IPSS (7). Due to a small number of variables fitted in the regression tree, no pruning was done. All tests were two sided with a level of significance set at *p* < 0.05, and R software environment for statistical computing and graphics (version 3.4.3) was used for all analyses.

## Results

### Baseline Patient Characteristics

Of the 397 patients, who were referred to our tertiary care laser center for HoLEP due to suspected BPH between 11/2017 and 07/2020, 83 patients (20.7%) underwent further examinations, based on suspicious prostate characteristics (Figure 1). In those patients, seven (8.7%) PCa cases were identified by prostate biopsy prior to BPH treatment with HoLEP (Table 1). Patients with PCa detection were in median older than patients with negative PCa detection in further examinations (72 vs. 66 years). Moreover, total PSA value and fPSA ratio were lower in the PCa detection group [6.7 ng/ml (IQR 4.7–11.3) and 16% (IQR 14–18)], relative to the negative PCa detection group prior to HoLEP [8.9 ng/ml (IQR 5.6–14.4) and 19% (IQR 15–28)]. These differences in patient and prostate characteristics were clinically meaningful, although not reaching statistical significance (all *p* > 0.05). No clinically meaningful or statistically significant differences were recorded in median PSA density (0.09 vs. 0.09 ng/ml/cc, *p* = 0.1) between both groups.

**Table 1:** Descriptive characteristics of 83 patients undergoing advanced prostate cancer evaluation prior to Holmium Laser Enucleation of the Prostate (HoLEP) for Benign Prostate Hyperplasia (BPH) treatment, due to suspicious prostate characteristics, stratified according to negative or prostate cancer detection.

**Variable**		**Overall *n* = 83**	**Negative PCa detection *n* = 76 (91.6%)**	**PCa detection *n* = 7 (8.4%)**	***p*-value**
Age, years	Median (IQR)	67 (61–72)	66 (61–72)	72 (68–74)	0.1
Prostate volume, cc	Median (IQR)	81 (65–120)	84 (65–121)	69 (65–78)	0.2
PSA, ng/ml	Median (IQR)	8.7 (5.4–14.2)	8.9 (5.6–14.4)	6.7 (4.7–11.3)	0.3
PSA ratio, %	Median (IQR)	18 (15–28)	19 (15–28)	16 (14–18)	0.5
PSA density, ng/ml/cc	Median	0.09 (0.07–0.15)	0.09 (0.07–0.15)	0.09 (0.07–0.11)	1
Family history of PCa	Yes	2 (2.4)	1 (1.3)	1 (14.3)	0.4
	No	81 (97.6)	75 (98.7)	6 (85.7)	
ASA status	I	7 (8.4)	7 (9.2)	0 (0)	0.2
	II	60 (72.3)	56 (73.7)	4 (57.1)	
	III	16 (19.3)	13 (17.1)	3 (42.9)	
Catheter before surgery	Yes	37 (44.6)	33 (43.4)	4 (57.1)	0.8
	No	46 (55.4)	43 (56.6)	3 (42.9)	
Q max, ml/s	Median (IQR)	10.0 (6.0–12.0)	8.5 (5.8–11.3)	15.4 (10.9–16.0)	0.3
Q average, ml/s	Median (IQR)	4.8 (4.0–5.5)	4.0 (4.0–5.0)	8.0 (8.0–8.0)	0.2
Residual urine, ml	Median (IQR)	90 (43–165)	95 (83–163)	15 (8–408)	0.4
BPH medication	None	24 (28.9)	22 (28.9)	2 (28.6)	0.8
BPH medication	alpha inhibitor	52 (62.7)	48 (63.2)	4 (57.1)	
	5–alpha reductase inhibitor/combination	7 (8.4)	6 (7.9)	1 (14.3)	
IPSS	Median (IQR)	20 (15–26)	21 (15–26)	12 (12–13)	0.02
DRE	Non-suspicious	80 (96.4)	76 (100)	4 (57.1)	<0.01
	suspicious	3 (3.6)	0 (0)	3 (42.9)	
Number of previous negative biopsies	0	61 (63.9)	50 (65.8)	3 (42.9)	0.2
	1	25 (30.1)	21 (27.6)	4 (57.1)	
	≥2	5 (6.0)	5 (6.6)	0 (0)	
Prostate biopsy within 1 year prior surgery	No	33 (39.8)	33 (43.4)	0 (0)	0.07
	Yes	50 (60.2)	43 (56.6)	7 (100)	
mpMRI within 1 year prior surgery	No	27 (32.5)	24 (31.6)	3 (42.9)	0.9
	Yes	56 (67.5)	52 (68.4)	4 (57.1)	
PIRADS in mpMRT prior surgery	PIRADS 1–2	22 (39.3)	22 (42.3)	0 (0)	0.01
	PIRADS 3	7 (12.5)	7 (13.5)	0 (0)	
	PIRADS 4–5	14 (25.0)	10 (19.2)	4 (100)	
	Unknown	13 (23.2)	13 (25.0)	0 (0)	
Location of PIRADS lesions in the prostate	Peripheral zone	20 (54.0)	18 (54.5)	2 (50.0)	0.8
	Transitional zone	12 (32.4)	11 (33.3)	1 (25.0)	
	Both	5 (13.5)	4 (12.1)	1 (25.0)	
Prostate cancer evaluation prior to surgery	mpMRI only	33 (39.8)	33 (43.4)	0 (0)	0.03
	Randomized biopsy	31 (37.3)	28 (36.8)	3 (42.9)	
	Fusion biopsy	19 (22.9)	15 (19.7)	4 (57.1)	

*PCa, Prostate cancer; IQR, Interquartile range; PSA, Prostate Specific Antigen; ASA, American Society of Anesthesia classification; Qmax, Maximum volume, Q average: Average volume; IPSS, International prostate symptom score; DRE, Digital rectal examination; mpMRI, multiparametric magnet resonance imaging; RP, Radical prostatectomy*.

### Differences in Bladder Outlet Obstruction Characteristics

The comparison between patients with and without malignant findings in advanced clinical PCa evaluation revealed important observations according to BOO characteristics (Table 1). First, IPSS was significantly lower in patients with PCa detection [12 (IQR 12–13)], relative to the control group [21 (15–26), *p* = 0.02]. Moreover, prostate volume was also lower in the PCa detection group [69 (IQR 65–78) vs. 84cc (65–121)], although not reaching statistical significance (*p* = 0.2). No clinically or statistically meaningful differences were observed according to uroflowmetry measurements (Qmax and Qaverage), indwelling catheter prior to planed BOO surgery, or BOO pharmacological medication.

### Diagnostic Characteristics of Patients With Prostate Cancer Detection in Advanced Clinical PCa Evaluation

Of the 83 patients with advanced clinical PCa evaluation, malignant findings were detected in seven (8.4%) patients. Family history of PCa was positive in 14.3% of those patients. Furthermore, DRE was suspicious in 42.9% of those patients, and 57.1% of those patients underwent mpMRI for PCa evaluation. In mpMRI, all patients had a highly suspicious PIRADS 4/5 lesion, which predominantly were located in the peripheral zone of the prostate. All patients with PIRADS 4/5 lesions in mpMRI underwent subsequent MRI-fusion biopsy afterward. No patient with PCa detection had a history of more than one negative biopsy. However, in patients with negative PCa detection prior to scheduling for HoLEP, 6.6% of patients had ≥2 prior negative prostate biopsies (Table 1).

### Prediction of Prostate Cancer Detection

**Figure 2:**
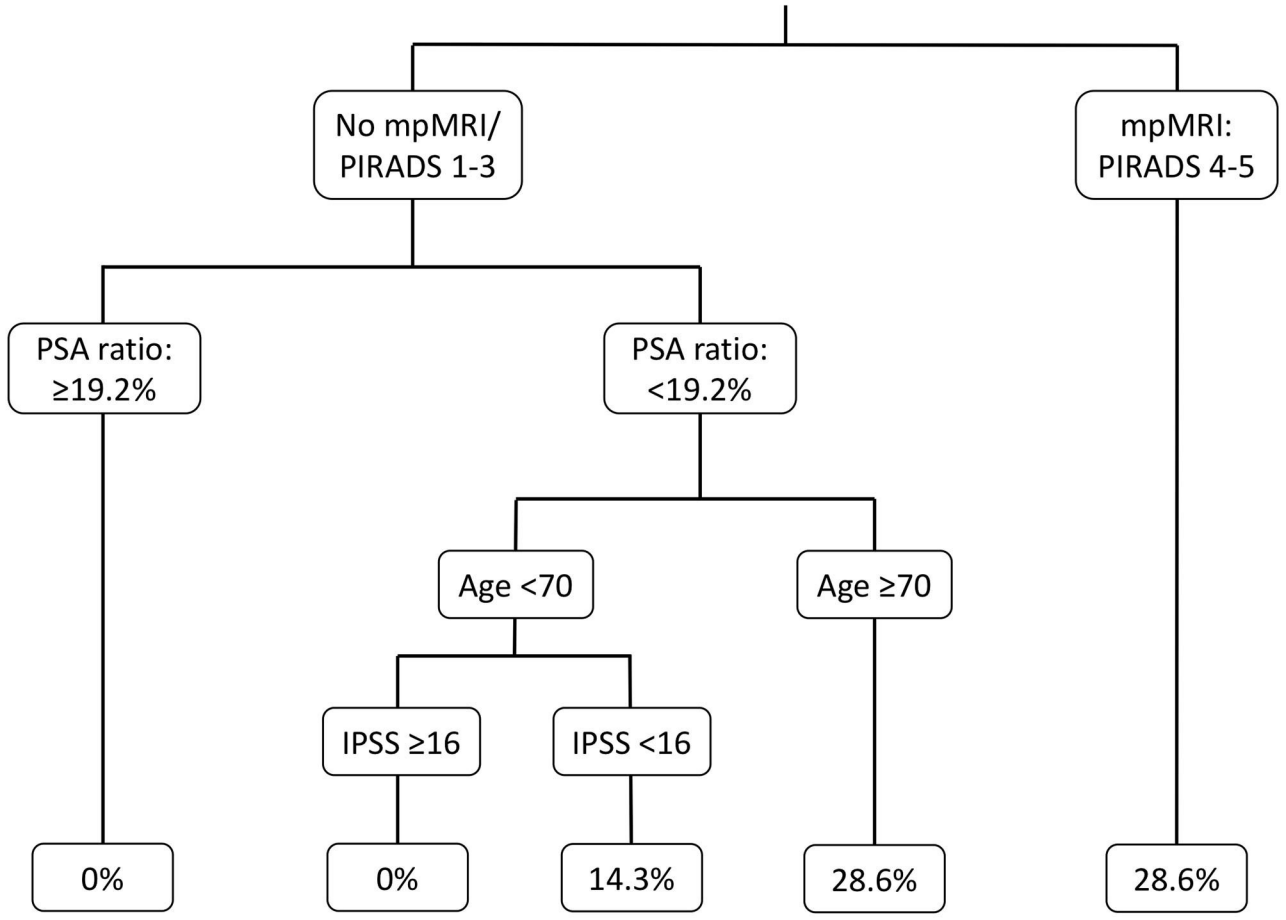
Binary regression tree depicting probability of positive prostate biopsy in patients with suspicious prostate characteristics, underwent further prostate cancer evaluation before Benign Prostate Hyperplasia treatment with Holmium Laser Enucleation of the Prostate (HoLEP). mpMRI, Multiparametric magnet resonance imaging; PSA, Prostate specific antigen; IPSS, International prostate symptom score.

In univariable regression models (Table 2), IPSS>16 [odds ratio (OR) 0.09, *p* = 0.04] and PIRADS 4/5 lesions (OR: 8.80, *p* < 0.01) represented predictors of PCa detection prior to BOO treatment. In multivariable regression models, PIRADS 4/5 lesions (OR: 9.91, *p* = 0.04) was an independent predictor for higher probability of PCa detection, while IPSS > 16 did not reach statistical significance (OR: 0.10, *p* = 0.052). Additionally, in the binary regression tree analyses (Figure 2), the resulting cut by the regression tree for the variables PIRADs score in mpMRI, fPSA ratio, age, and IPSS were, respectively, PIRADS 4/5 vs. no mpMRI/PIRADS1–3, <19.2% vs. ≥19.2%, ≥70 vs. <70 years, and <16 vs. ≥16, with an accuracy of 0.57. PI-RADS cutoff represented the root node of the decision tree, corroborating its possible impact in clinical decision making.

**Table 2:** Univariable and multivariable logistic regression model predicting prostate cancer detection in BPH patients, who underwent advanced prostate cancer evaluation prior to scheduled Holmium Laser Enucleation of the Prostate (HoLEP).

	**Univariable**			**Multivariable**		
	**OR**	**CI 2.5 −97.5%**	***P*-value**	**OR**	**CI 2.5 −97.5%**	***P*-value**
Age	1.09	0.98–1.24	0.1			
Prostate volume	0.99	0.97–1.01	0.4			
Previous biopsies	Ref. (1.0)	–	–	–	–	–
Biopsy naive	0.39	0.08–1.89	0.2	–	–	–
Catheter prior to surgery	1.74	0.35–9.33	0.5			
PSA	0.92	0.76–1.03	0.3	–	–	–
fPSA/PSA ratio	0.98	0.89–1.02	0.6	–	–	–
No mpMRI/PIRADS 1–3	Ref. (1.0)	–	–	–	–	–
PIRADS 4–5	8.80	1.71–50.65	<0.01	9.91	1.18–89.11	0.036
IPSS ≤16	Ref. (1.0)	–	–	–	–	–
IPSS >16	0.09	0.004–0.69	0.039	0.10	0.005–0.79	0.052

*fPSA, free Prostate specific antigen; PSA, Prostate specific antigen; mpMRI, multiparametric magnetic resonance imaging; IPPS, International prostate symptom score*.

After PCa detection prior to scheduling for HoLEP and BOO treatment, a change in treatment occurred in 100% of patients toward radical prostatectomy, of whom 0, 14.3, 57.1, and 28.6% exhibited pT2a, pT2b, pT2c, or ≥pT3 stage. Pathological Gleason score 6, 7, and 8–10 occurred in 28.6, 57.1, and 14.3% of those patients, respectively (Table 3).

**Table 3:** Pathological characteristics of 7 patients, who underwent radical prostatectomy due prostate cancer detection after advanced prostate cancer evaluation prior to scheduled Holmium Laser Enucleation of the prostate (HoLEP) due to symptomatic Benign Prostate Syndrome (BPS).

**Variable**		**RP** ***n* = 7**
Pathological T stage	pT2a	0 (0)
	pT2b	1 (14.3)
	pT2c	4 (57.1)
	pT3a	1 (14.3)
	pT3b	1 (14.3)
	pT4	0 (0)
Pathological Gleason score	GS 6	2 (28.6)
	GS 7	4 (57.1)
	GS 8–10	1 (14.3)

*RP, Radical prostatectomy; GS, Gleason score*.

In patients with negative PCa detection in preoperative advanced clinical PCa evaluation, incidental PCa after HoLEP occurred in 11.8%, of whom 88.9% harbored pT1a and 11.1% pT1b stage. Gleason score 6, 7, and 8–10 occurred in 77.8, 22.2, and 0% in those patients, respectively (Table 4).

**Table 4:** Pathological characteristics of 76 patients, who underwent Holmium Laser Enucleation of the Prostate (HoLEP) due to symptomatic Benign Prostate Hyperplasia (BPH) after advanced prostate cancer evaluation prior to HoLEP.

**Variable**		**HoLEP *n =* 76**
Incidental prostate cancer	No	67 (88.2)
	Yes	9 (11.8)
Pathological T stage	T1a	8 (10.5)
	T1b	1 (1.3)
Pathological Gleason score	GS 6	7 (77.8)
	GS 7	2 (22.2)
	GS 8–10	0 (0)

*PCa, Prostate cancer; GS, Gleason score*.

## Discussion

We aimed to investigate the characteristics of patients with clinical BOO scheduled for HoLEP, who underwent advanced clinical PCa evaluation using mpMRI and/or prostate biopsies. We relied on our institutional database encompassing 397 patients and hypothesized that patients who were diagnosed with PCa in advanced clinical evaluation may be identified by standard clinical and PSA-based examinations. We observed several noteworthy findings.

First, mpMRI might represent a valid tool for PCa detection in patients with suspicious basic PCa evaluation findings such as elevated total PSA or low fPSA ratio. In our cohort, an mpMRT was performed in two thirds of all patients after suspicious findings in basic PCa detection. Here, PIRADs 4/5 lesions were found in one fourth. Of those, PCa was found in one fourth after mpMRI fusion biopsy of the prostate. Although this rate seems rather low, it should be taken into account that more than one third of the patients scheduled for HoLEP presented with a history of one or more negative biopsies (8–10). In contrast to our findings, Preisser et al. observed a detection rate of 71.6% in biopsy naïve, 50.9% in patients after one negative biopsy, and 43.5% after two negative biopsies for mpMRI fusion biopsy (11). Moreover, it is noteworthy that no patient with a history of more than one negative prostate biopsies was diagnosed with malignant findings in advanced clinical PCa evaluation or after HoLEP. Additionally, no patient with mpMRI PIRADs one to three lesions was diagnosed with malignant findings. In consequence, patients with a history of more than one previous negative prostate biopsies or finding of PIRADS one to three lesions in mpMRI invasive PCa evaluation with prostate biopsy might be omitted prior to scheduling for HoLEP. Thus, mpMRI does not only identify patients with suspicious lesions for PCa but also helps to avoid unnecessary prostate biopsies.

Second, standard PCa evaluation using (total) serum PSA measurement cannot reliably identify patients with underlying PCa who are presenting with clinical BOO. Potentially, enlargement of the prostate, urinary retention, or indwelling transurethral catheters as well as others represent confounding factors limiting the validity of this otherwise helpful clinical tool (4, 12–16). Interestingly, also after accounting for prostate volume using PSA density, no clinical or statistically meaningful differences between patients with malignant and benign BOO were observed.

Third, DRE and fPSA ratio might represent simple and effective basic examinations in PCa evaluation in patients with clinical BOO. Although, fPSA ratio was not confirmed with a statistically significant association of PCa detection in uni- or multivariable analyses, the confidence interval and the central tendency suggested that this was caused by too few observations. Based on the decision tree analyses, we observed that the fPSA ratio cut at <19.2% predicts PCa detection at its best in our model and almost agrees with previously described fPSA ratio cutoffs and current guidelines recommendations (4, 17–20). Nonetheless, it is noteworthy that, for example, the PSA ratio should be only applied and taken into account below an absolute PSA value <10 ng/ml (4). Moreover, decision making should not be based only on PSA ratio alone due to low sensitivity and specificity rates (17, 21, 22). As for DRE, all patients with suspicious DRE had PCa detection after biopsy. Therefore, statistical evaluation of DRE status using logistic regression analyses was not valid. Nevertheless, our results corroborate current clinical practice, to pursuit biopsy confirmation in patients with suspicious finding in DRE.

Fourth, patients with underlying PCa and patients with benign BOO differ in regard of LUTS characteristics. For example, IPSS was significantly lower in the group with PCa detection (12 vs. 21), compared with the cohort with negative PCa detection. Conversely, minor, if any, differences were recorded for other BOO characteristics such as uroflowmetry, catheter usage, or BOO medication. Nonetheless, our results suggest that a lower IPSS, as a proxy for severity of LUTS, should make clinicians be aware for thorough PCa evaluation, since the urgency for fast HoLEP scheduling is not given.

Finally, advanced clinical PCa evaluation of patients before scheduling for HoLEP was not without clinical impact: Preoperative PCa detection led to a change in treatment in all patients toward radical prostatectomy. Of those patients, 28.6% harbored locally advanced PCa (≥pT3 stage), and, respectively, 57.1 and 14.3% Gleason scores 7 and 8–10. To the contrary, in patients with negative PCa detection prior to HoLEP, 11.8% exhibited incidental PCa, of whom the majority harbored pT1a stage (88.9%) and, respectively, 77.8 and 22.2% Gleason scores 6 and 7, respectively. Interestingly, in comparison to our entire HoLEP database, the rate of incidental PCa was still higher in the cohort of patients with advanced clinical preoperative PCa evaluation using mpMRI and/or prostate biopsy (11.8 vs. 8.1%, data not shown). However, these numbers were in an agreement with current literature of incidental prostate cancer (23). Moreover, patients with PCa detection prior to planed HoLEP exhibited worse pathological characteristics, relative to incidental PCa patients. Thus, preoperative advanced clinical PCa evaluation in patients with clinical BOO helps in selecting patients with adverse pathological characteristics for curative treatment.

Our study has several limitations. First, our study is based on retrospective analyses. As such, PCa evaluation prior to HoLEP is subjected to a selection bias. Prospective evaluation is mandatory to confirm our observational results. Second, the limited number of observations impairs statistical significance in our analyses, especially in some patient characteristics. Third, though all mpMRI examinations were primarily performed and read by an experienced radiologist and confirmed by a board-certified radiologist, interobserver variability cannot be ruled out. Moreover, the exact value of the mpMRI in BOO patients would only be completely investigable if all patients would have undergone fusion biopsy after mpMRI. Finally, the number of variables in our multivariable logistic regression model was limited by the observations for the outcome variable, and therefore, the adjustment may lack important variables. Ideally, multi-institutional studies investigating a larger cohort are needed to further validate our findings.

Nevertheless, our results confirm that advanced clinical PCa evaluation with mpMRI and/or prostate biopsy is worthwhile in patients with clinical BOO and suspicious or inconclusive findings in standard clinical PCa evaluation prior to HoLEP. While standard clinical PCa evaluation using only total serum PSA is not able to identify patients at risk for PCa finding, PIRADS 4/5 lesions in mpMRI represent an independent risk factor. Moreover, pathological digital rectal examination, a low fPSA ratio, and a low IPSS might also help to identify patients with underlying PCa. Multicenter studies may promote the understanding of the exact role of mpMRI in the evaluation of BPH patients scheduled for HoLEP.

## Data Availability Statement

The raw data supporting the conclusions of this article will be made available by the authors, without undue reservation.

## Ethics Statement

The studies involving human participants were reviewed and approved by Ethic committee University Hospital Frankfurt, approval number SUG-9-2018. Written informed consent for participation was not required for this study in accordance with the national legislation and the institutional requirements.

## Author Contributions

MW, PK, LK, FC, PM, and AB did the writing/editing of the manuscript. MW, MNW, PK, FC, and AB were in charge of the protocol/project development. MW, LG, FP, MD, SB, and BB did the data collection or management. MW, LG, LT, CH, SB, BB, and AB analyzed the data. MD, PG, and SA made academical input. PG and SA also edited the manuscript. All authors contributed to the article and approved the submitted version.

## Conflict of Interest

The authors declare that the research was conducted in the absence of any commercial or financial relationships that could be construed as a potential conflict of interest.
